# EGFR‐Induced and c‐Src‐Mediated CD47 Phosphorylation Inhibits TRIM21‐Dependent Polyubiquitylation and Degradation of CD47 to Promote Tumor Immune Evasion

**DOI:** 10.1002/advs.202206380

**Published:** 2023-08-04

**Authors:** Linyong Du, Zhipeng Su, Silu Wang, Ying Meng, Fei Xiao, Daqian Xu, Xinjian Li, Xu Qian, Su Bin Lee, Jong‐Ho Lee, Zhimin Lu, Jianxin Lyu

**Affiliations:** ^1^ Key Laboratory of Laboratory Medicine Ministry of Education of China School of Laboratory Medicine and Life Science Wenzhou Medical University Wenzhou Zhejiang 325035 China; ^2^ Department of Neurosurgery First Affiliated Hospital of Wenzhou Medical University Wenzhou Medical University Wenzhou Zhejiang 325000 China; ^3^ Key Laboratory of Diagnosis and Treatment of Severe Hepato‐Pancreatic Diseases of Zhejiang Province The First Affiliated Hospital of Wenzhou Medical University Wenzhou Medical University Wenzhou Zhejiang 325000 China; ^4^ Zhejiang Provincial Key Laboratory of Pancreatic Disease of The First Affiliated Hospital Institute of Translational Medicine Zhejiang University School of Medicine Zhejiang University Hangzhou Zhejiang 310029 China; ^5^ Cancer Center Zhejiang University Hangzhou Zhejiang 310029 China; ^6^ CAS Key Laboratory of Infection and Immunity CAS Center for Excellence in Biomacromolecules Institute of Biophysics Chinese Academy of Sciences Beijing 100101 China; ^7^ Department of Nutrition and Food Hygiene Center for Global Health School of Public Health Nanjing Medical University Nanjing Jiangsu 211166 China; ^8^ Department of Health Sciences The Graduate School of Dong‐A University Busan 49315 Republic of Korea; ^9^ People's Hospital of Hangzhou Medical College Hangzhou Zhejiang 310014 China

**Keywords:** CD47, c‐Src, epidermal growth factor receptor, immune evasion, polyubiquitylation, TRIM21, tumor cells

## Abstract

Tumor cells often overexpress immune checkpoint proteins, including CD47, for immune evasion. However, whether or how oncogenic activation of receptor tyrosine kinases, which are crucial drivers in tumor development, regulates CD47 expression is unknown. Here, it is demonstrated that epidermal growth factor receptor (EGFR) activation induces CD47 expression by increasing the binding of c‐Src to CD47, leading to c‐Src‐mediated CD47 Y288 phosphorylation. This phosphorylation inhibits the interaction between the ubiquitin E3 ligase TRIM21 and CD47, thereby abrogating TRIM21‐mediated CD47 K99/102 polyubiquitylation and CD47 degradation. Knock‐in expression of CD47 Y288F reduces CD47 expression, increases macrophage phagocytosis of tumor cells, and inhibits brain tumor growth in mice. In contrast, knock‐in expression of CD47 K99/102R elicits the opposite effects compared to CD47 Y288F expression. Importantly, CD47‐SIRPα blockade with an anti‐CD47 antibody treatment significantly enhances EGFR‐targeted cancer therapy. In addition, CD47 expression levels in human glioblastoma (GBM) specimens correlate with EGFR and c‐Src activation and aggravation of human GBM. These findings elucidate a novel mechanism underlying CD47 upregulation in EGFR‐activated tumor cells and underscore the role of the EGFR‐c‐Src‐TRIM21‐CD47 signaling axis in tumor evasion and the potential to improve the current cancer therapy with a combination of CD47 blockade with EGFR‐targeted remedy.

## Graphical abstract

CD47 is frequently overexpressed in tumors and promotes tumor cell immune evasion. It is demonstrated that epidermal growth factor receptor (EGFR) activation induces c‐Src‐mediated CD47 phosphorylation. This phosphorylation inhibits the interaction between the TRIM21 and CD47, thereby abrogating TRIM21‐mediated CD47 polyubiquitylation and degradation. Importantly, CD47 blockade significantly enhances EGFR‐targeted cancer therapy in an animal model.

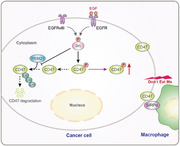

## 1 Introduction

The innate immune system provides the first line of defense against infections and malignant cell transformations.^[^ ^1^ ^]^ Through phagocytosis, antigen‐presenting cells (APCs), including monocytes, dendritic cells, and macrophages, are crucial parts of the innate immune system and are able to capture and eliminate transformed malignant cells. In addition, APCs function as a bridge to the adaptive immune system and present tumor‐derived antigens to prime T cells and activate downstream adaptive immune responses.^[^ ^1^ ^]^ However, tumor cells often overexpress immune checkpoint proteins for immune evasion. Integrin‐associated protein (IAP or CD47), a glycosylated five‐transmembrane protein, is frequently overexpressed in hematologic and solid tumors, allowing tumor cells to evade innate immune surveillance.^[^ ^2^ ^]^ CD47 binds to and activates signal regulatory protein α (SIRPα), an inhibitory protein expressed on the surface of myeloid cells including all types of macrophages. Activation of SIRPα initiates a signaling cascade that inhibits the phagocytic activity of macrophages, and CD47, therefore, functions as an anti‐phagocytic or “don't eat me” signal to avoid self‐elimination by phagocytes.^[^ ^3^ ^]^ The administration of CD47‐blocking monoclonal antibodies for cancer treatment has been investigated in multiple clinical trials.^[^ ^2^ ^]^

CD47 expression can be regulated at the transcriptional level by multiple transcription factors, including signal transducer and activator of transcription 3 (STAT3),^[^ ^4^ ^]^ β‐catenin–transcription Factor 4 (TCF4),^[^ ^5^ ^]^ hypoxia‐inducible factor 1 (HIF‐1),^[^ ^6^ ^]^ and c‐Myc.^[^ ^7^ ^]^ However, whether CD47 expression is regulated at the posttranslational level, especially in response to tumor‐frequently occurred receptor tyrosine kinases (RTKs) lesions, thereby contributing to tumor immune evasion remains unclear. In addition, whether intervening oncogenic signaling‐regulated CD47 expression can sensitize RTK‐targeted therapy is unknown.

In this study, we demonstrated that activation of epidermal growth factor (EGF) receptor (EGFR) results in c‐Src‐mediated CD47 Y288 phosphorylation and subsequent abrogation of the binding of ubiquitin E3 ligase tripartite motif‐containing protein 21 (TRIM21) to CD47, thereby abrogating TRIM21‐mediated CD47 K99/102 polyubiquitylation and CD47 degradation. CD47 Y288F knock‐in expression reduced CD47 expression, increased macrophage phagocytosis of tumor cells, and inhibited brain tumor growth in mice. Importantly, CD47 blockade enhanced EGFR‐targeted cancer therapy.

## 2 Results

### 2.1 CD47 is Highly Expressed in GBM and Correlates with a Poor Prognosis in GBM Patients

**Figure 1:**
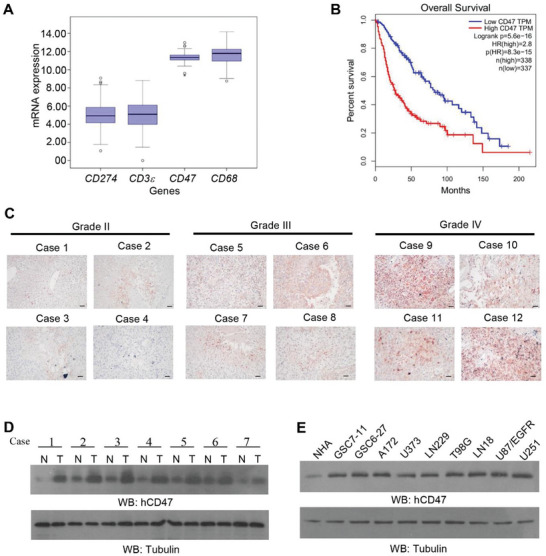
CD47 is highly expressed in GBM and correlates with a poor prognosis in GBM patients. A) Relative mRNA expression levels of the indicated genes were analyzed in the TCGA cohort of GBM (*n* = 162). B) Kaplan–Meier 18‐year overall survival analysis comparing CD47 high‐ and low‐expressing patients in the TCGA GBM cohort. The CD47 high and low groups were separated by the median expression. Significance was determined with the log‐rank test. *p* = 5.6 × 10^−16^; HR, 2.8. C) IHC staining of 75 human glioma specimens of different grades (II–IV) was performed with an anti‐CD47 antibody. Representative images of IHC staining from the specimens are shown. Scale bar, 100 µm. D) Immunoblotting analysis of CD47 protein expression in paired tumor‐adjacent normal tissues (N) and human GBM specimens (T). E) The protein expression levels of CD47 in NHA cells and the indicated human GBM and glioma stem cells (GSCs) were determined by immunoblotting analyses.

Malignant gliomas, especially grade IV glioblastoma (GBM), progress rapidly with a limited response to current therapies.^[^ ^8^ ^]^ Analyses of The Cancer Genome Atlas (TCGA, https://www.cancer.gov/ccg/research/genome‐sequencing/tcga) dataset (*n* = 162) showed that CD47 and the macrophage marker CD68 are highly expressed in GBM tissues compared to CD274 (encoding PD‐L1) and CD3e (encoding a component of the TCR‐CD3 complex on the T‐lymphocyte cell membrane) (**Figure** **1**A). These results suggested that the expression of CD47 is high in GBM tissues in which macrophages are highly infiltrated.

Analyses of glioma patient survival showed that the patients with high CD47 expression had a shorter survival time than those with low expression of CD47 (Figure 1B and Figure S1A, Supporting Information). Immunohistochemical (IHC) analyses of 75 human glioma specimens with different grades (II–IV) showed that CD47 expression levels were positively associated with grade levels of glioma (Figure 1C and Figure S1B, Supporting Information). In addition, immunoblotting analyses revealed that CD47 protein expression was higher in GBM tissues than in paired adjacent normal tissues (Figure 1D). Consistently, human GBM (Figure 1E), including GSC7‐11 and GSC6‐27 primary GBM cells, or mouse glioma (Figure S1C, Supporting Information) cells, exhibited higher expression levels of CD47 than either normal human astrocytes (NHA) or mouse normal brain tissues, respectively. These results indicated that CD47 is highly expressed in GBM cells and correlated with a poor prognosis in GBM patients.

### 2.2 EGFR Activation Induces CD47 Expression Independent of its Transcription and Translation

**Figure 2:**
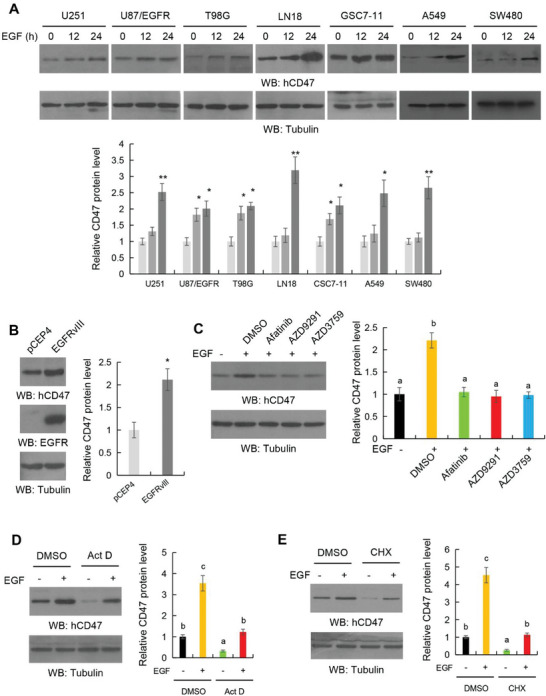
EGFR activation induces CD47 protein upregulation. A) The indicated human tumor cells were serum‐starved for 12 h and then stimulated with EGF (100 ng mL^−1^) for the indicated periods of time. Immunoblot analyses were performed with the indicated antibodies (top panel). Quantification of relative CD47 protein levels is shown (bottom panel). B) U87 cells were stably transfected with plasmids expressing control vector or EGFRvIII. Immunoblot analyses were performed with the indicated antibodies (left panel). Quantification of relative CD47 protein levels is shown (right panel). C) Serum‐starved U251 cells were stimulated with EGF (100 ng mL^−1^) for 24 h in the presence or absence of the indicated EGFR inhibitors. Immunoblot analyses were performed (left panel). Quantification of relative CD47 protein levels is shown (right panel). D) Serum‐starved U251 cells were stimulated with or without EGF (100 ng mL^−1^) for 24 h in the presence of DMSO or actinomycin D (1 µg mL^−1^). Immunoblot analyses were performed (left panel). Quantification of relative CD47 protein levels is shown (right panel). E) Serum‐starved U251 cells were stimulated with or without EGF (100 ng mL^−1^) for 24 h in the presence of DMSO or CHX (100 µg mL^−1^). Immunoblot analyses were performed (left panel). Quantification of relative CD47 protein levels is shown (right panel). The results represent the means ± SD; ANOVA two‐way test. Different letters indicate significant differences (*p* < 0.05); *, *p* < 0.05, **, *p* < 0.01 on the basis of Student's *t*‐test.

Overexpression or mutation of EGFR frequently occurs in human GBM.^[^ ^8^, ^9^ ^]^ To examine whether EGFR activation regulates CD47 expression, we treated a panel of human GBM cells, including U251, U87/EGFR, T98G, LN18, and GSC7‐11 cells, A549 lung cancer cell, SW480 colorectal cancer cell (**Figure** **2**A), and mouse glioma cells, including GL261 and CT‐2A, (Figure S2A, Supporting Information) with EGF. Immunoblotting analyses showed that EGF induced CD47 expression in a time‐dependent manner (Figure 2A and Figure S2A, Supporting Information) while flow cytometry analyses revealed that EGF increased CD47 expression on the plasma membrane (Figure S2B, Supporting Information). In addition, an increase in CD47 expression in U87 human GBM cells was also observed by ectopically expressing an activated EGFRvIII mutant, which is frequently detected in human GBM (Figure 2B).^[^ ^8^ ^]^ Pretreatment of U251 human GBM (Figure 2C) and CT‐2A mouse glioma (Figure S2C, Supporting Information) cells with the EGFR inhibitors afatinib, AZD9291, or AZD3759 blocked EGF‐induced CD47 expression. Nevertheless, mRNA analyses revealed that EGFR activation did not obviously alter CD47 mRNA expression (Figure S2D–F, Supporting Information). In addition, pretreatment with actinomycin D (Act D) (Figure 2D) and cycloheximide (CHX) (Figure 2E), which inhibit gene transcription and translation, respectively, did not reduce the fold induction of CD47 expression upon EGF treatment. These results suggested that EGFR activation induces CD47 expression in a posttranslational mechanism‐dependent manner.

### 2.3 c‐Src Activation Increases CD47 Expression by Stabilizing CD47

**Figure 3:**
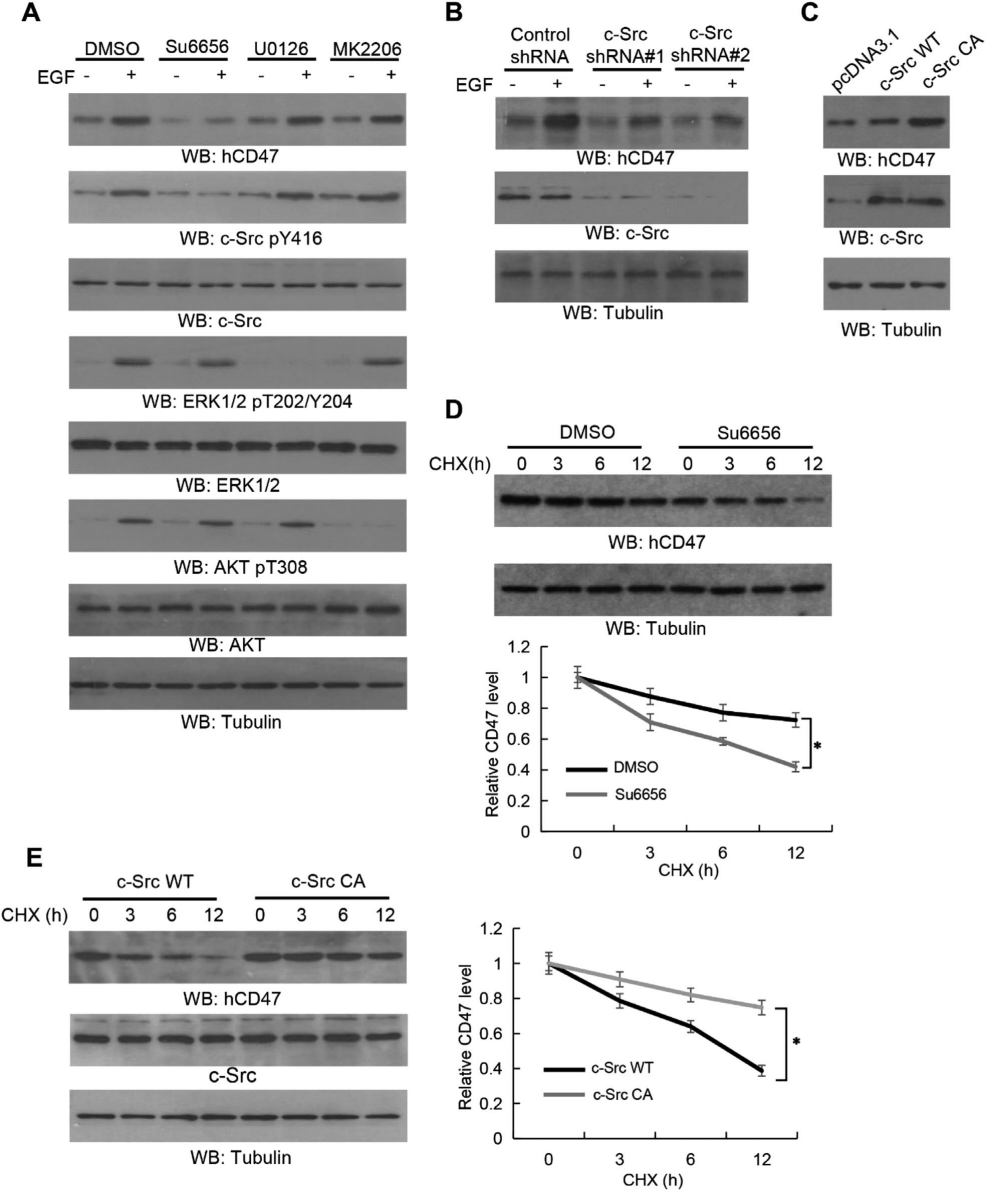
c‐Src activation induces CD47 protein upregulation in response to EGFR activation. Immunoblot analyses were performed with the indicated antibodies (A–E). A) Serum‐starved U251 cells were pretreated with the indicated inhibitors for 2 h and then stimulated with EGF (100 ng mL^−1^) for 24 h. B) Serum‐starved U251 cells with stable expression of different shRNAs against c‐Src or a control shRNA were stimulated with or without EGF (100 ng mL^−1^) for 24 h. C) U251 cells were transfected with a control vector, wild‐type (WT) c‐Src, or an active c‐Src (CA) for 48 h. D) U87/EGFRvIII cells were pretreated with DMSO or Su6656 (4 µm) for 2 h and then treated with CHX (100 µg mL^−1^) for the indicated periods of time. Quantification of relative CD47 protein levels is shown (bottom panel). E) U251 cells stably expressed c‐Src WT or c‐Src CA were treated with CHX (100 µg mL^−1^) for the indicated periods of time. Quantification of relative CD47 protein levels is shown (right panel).

To further delineate the mechanism underlying EGF‐induced CD47 expression, we treated U251 cells with Su6656, U0126, and MK‐2206, which inhibited EGF‐induced activation of c‐Src, ERK, and AKT, respectively (**Figure** **3**A). Notably, only Su6656 treatment attenuated EGF‐induced CD47 expression. In line with these results, depletion of c‐Src by expressing its shRNA in U251 (Figure 3B) and U87/EGFRvIII (Figure S3A, Supporting Information) cells reduced CD47 expression in the presence or absence of EGF treatment, whereas expression of an active c‐Src (c‐Src CA) mutant substantially increased CD47 expression in U251 (Figure 3C) and EGFR‐overexpressing U87 (U87/EGFR) (Figure S3B, Supporting Information) cells. Correspondingly, depletion of c‐Src or Su6656 treatment reduced the half‐life of CD47 in U87/EGFRvIII cells (Figure 3D and Figure S3C, Supporting Information), whereas overexpression of the active c‐Src CA mutant prolonged the CD47 half‐life in U251 cells (Figure 3E). These results indicated that activation of c‐Src induced by EGFR activation increases CD47 expression by stabilizing CD47.

### 2.4 EGFR‐Activated c‐Src Binds to CD47, Phosphorylates CD47 at Y288, and Subsequently Upregulates CD47 Stability by Inhibiting CD47 Polyubiquitylation

**Figure 4:**
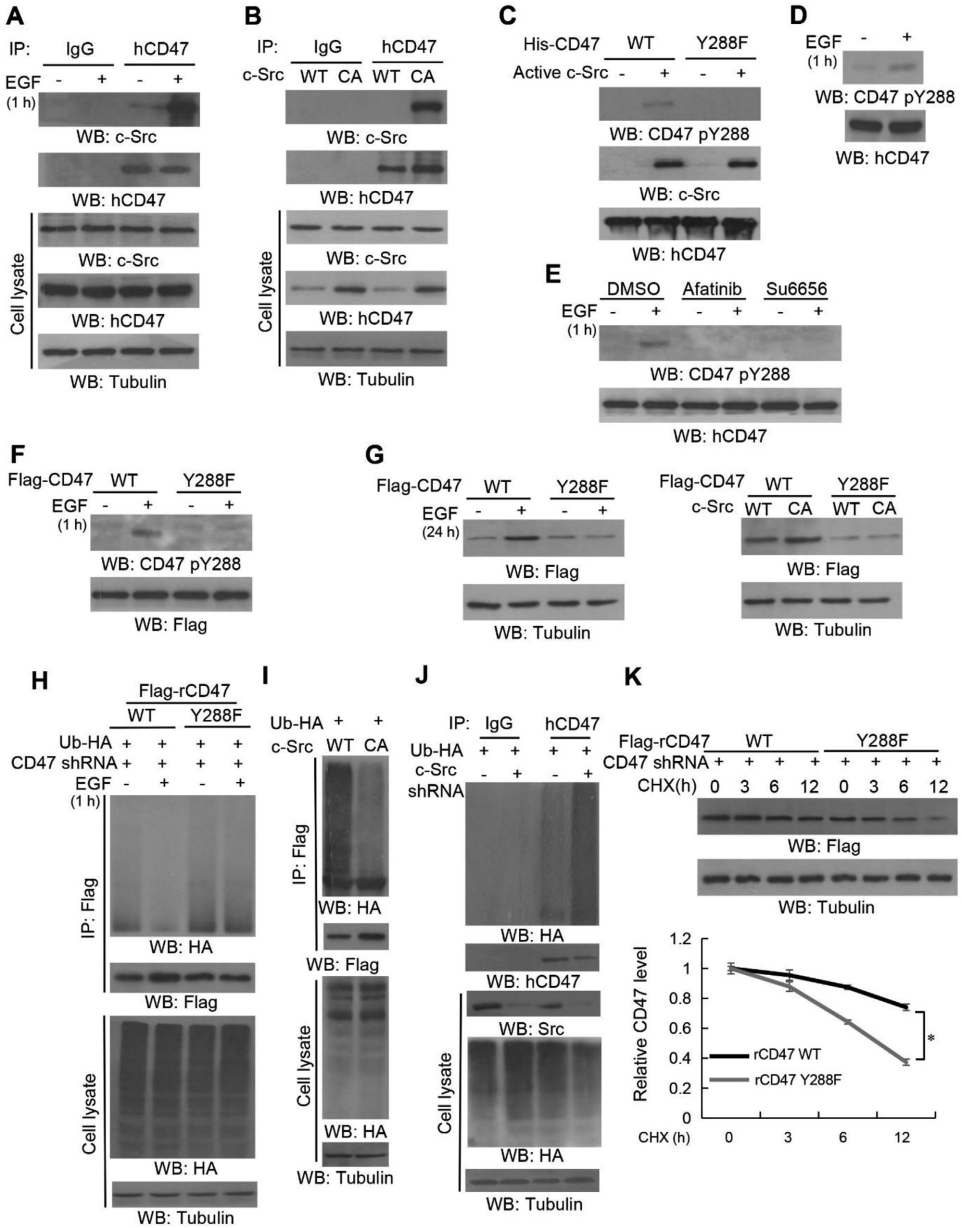
c‐Src binds to and phosphorylates CD47 at Y288, which subsequently upregulates CD47 stability by inhibiting CD47 polyubiquitylation. Immunoblotting analyses were performed with the indicated antibodies (A–K). A) Serum‐starved U251 cells were stimulated with or without EGF (100 ng mL^−1^) for 1 h. Endogenous CD47 was immunoprecipitated. B) U251 cells were transfected with c‐Src WT or c‐Src CA for 48 h. Endogenous CD47 was immunoprecipitated. C) In vitro kinase assays were performed by mixing purified WT His‐CD47 or His‐CD47 Y288F with or without active c‐Src. D) Serum‐starved U251 cells were stimulated with or without EGF (100 ng mL^−1^) for 1 h. E) Serum‐starved U251 cells were stimulated with or without EGF (100 ng mL^−1^) for 1 h in the presence or absence of the indicated inhibitors. F) HEK293T/EGFR cells were transiently transfected with WT Flag‐CD47 or the indicated Flag‐tagged mutants and stimulated with or without EGF (100 ng mL^−1^) for 24 h. G) HEK293T/EGFR cells were transiently transfected with WT Flag‐CD47 or the indicated Flag‐tagged mutants and stimulated with or without EGF (100 ng mL^−1^) for 24 h (left panel) or transiently co‐transfected with WT Flag‐CD47 or the indicated Flag‐tagged mutants and wild‐type or c‐Src CA for 48 h (right panel). H) CD47 knockdown U251 cells with reconstituted expression of WT Flag‐rCD47 or Flag‐rCD47 Y288F mutant were transfected with HA‐Ub and then stimulated with or without EGF (100 ng mL^−1^) for 60 min. MG132 (10 µm) was added to the cells 6 h before they were harvested with guanidine‐HCl‐containing buffer. Immunoprecipitation of Flag was performed with an anti‐Flag antibody. I) HEK293T/EGFR cells were co‐transfected with c‐Src WT or c‐Src CA, CD47‐Flag, and HA‐Ub. The cells were harvested with a guanidine‐HCl‐containing buffer. Immunoprecipitation was performed with an anti‐Flag antibody. J) U87/EGFRvIII cells were co‐transfected with control shRNA, c‐Src shRNA, or HA‐Ub. MG132 (10 µm) was added to the cells 6 h before they were harvested with guanidine‐HCl‐containing buffer. Immunoprecipitation was performed with an anti‐CD47 antibody. K) CD47‐depleted U87/EGFRvIII cells with reconstituted expression of WT Flag‐rCD47 or Flag‐rCD47 Y288F mutant were treated with CHX (100 µg mL^−1^) for the indicated periods of time. Quantification of relative Flag (rCD47) protein levels is shown (bottom panel).

To examine the relationship between c‐Src and CD47, we performed coimmunoprecipitation analyses and showed that EGF treatment (**Figure** **4**A) or expression of the active c‐Src CA mutant (Figure 4B) induced the binding of c‐Src to CD47, and the EGF‐induced binding was inhibited by treatment with EGFR inhibitors (Figure S4A, Supporting Information). Mass spectrum analyses of immunoprecipitated CD47 from EGF‐stimulated U251 cells showed that CD47 Y288 (Figure S4B, Supporting Information), an amino acid conserved in different species (Figure S4C, Supporting Information), was phosphorylated. An in vitro phosphorylation assay by mixing purified bacterial‐expressed activated c‐Src with purified bacterial‐expressed wild‐type (WT) His‐CD47 or His‐CD47 Y288F showed that activated c‐Src phosphorylated WT His‐CD47, but not His‐CD47 Y288F (Figure 4C), as detected by a specificity‐validated CD47 phospho‐Y288 (pY288) antibody (Figure S4D, Supporting Information). In addition, EGF treatment induced CD47 Y288 phosphorylation in both U251 (Figure 4D) and U87/EGFR (Figure S4D, Supporting Information) cells, and this phosphorylation was abrogated by treatment with the EGFR inhibitor afatinib and the c‐Src inhibitor Su6656 (Figure 4E) or expression of the CD47 Y288F mutant (Figure 4F). Notably, CD47 Y286F, which was expressed in endogenous CD47‐depleted mouse CT‐2A cells, showed resistance to EGF‐induced upregulation (Figure S4E, Supporting Information). Similarly, CD47 Y288F was resistant to EGF‐ or activated c‐Src‐induced upregulation of CD47 in human 293T/EGFR cells (Figure 4G). These results indicated that EGFR‐activated c‐Src binds to CD47, phosphorylates CD47 at Y288, and subsequently upregulates CD47 expression.

To examine whether ubiquitylation is involved in CD47 stability, we treated U251 cells with EGF (Figure 4H) or expressed the active c‐Src CA mutant (Figure 4I) and showed that both approaches reduced CD47 polyubiquitylation. c‐Src depletion (Figure 4J and Figure S4F, Supporting Information) or expression of CD47 Y288F (Figure 4H) increased CD47 polyubiquitylation. In addition, CD47 Y288F had a much shorter half‐life than its WT counterpart in U87/EGFRvIII (Figure 4K) and 293T (Figure S4G, Supporting Information) cells. These results indicated that c‐Src‐mediated CD47 Y288 phosphorylation inhibits CD47 polyubiquitylation and degradation.

### 2.5 CD47 Y288 Phosphorylation Inhibits TRIM21‐Mediated CD47 K99/102 Polyubiquitylation and CD47 Degradation

**Figure 5:**
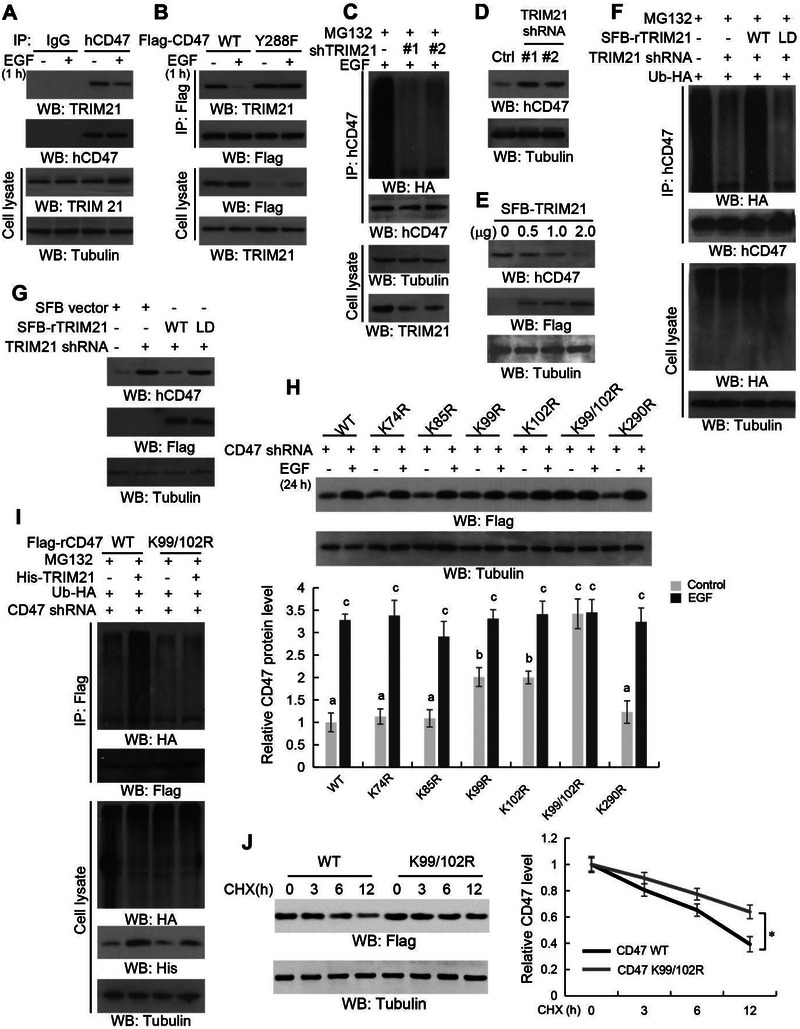
CD47 Y288 phosphorylation inhibits TRIM21‐mediated CD47 K99/102 polyubiquitylation and CD47 degradation. Immunoblotting analyses were performed with the indicated antibodies (A–J). A) U251 cells were treated with or without EGF (100 ng mL^−1^) for 1 h. Immunoprecipitation was performed with an anti‐CD47 antibody. B) HEK293T/EGFR cells were transiently transfected with Flag‐CD47 or Flag‐CD47 Y288F. The cells were treated with or without EGF (100 ng mL^−1^) for 1 h. Immunoprecipitation was performed with anti‐Flag beads. C) U251 cells stably expressing the indicated TRIM21 shRNAs or a control shRNA were transfected with HA‐Ub. MG132 (10 µm) was added to the cells 6 h before they were harvested with guanidine‐HCl‐containing buffer. Immunoprecipitation with an anti‐CD47 antibody was performed. D) U251 cells were stably expressed with the TRIM21 shRNAs or a control shRNA. TRIM21 shRNA#1 was used for the subsequent experiments. E) U251 cells were transiently transfected with the indicated amounts of SFB‐TRIM21 for 48 h and then the cells were collected for immunoblotting. F) U251 cells stably expressing TRIM21 shRNA or control shRNA with or without reconstituted expression of WT SFB‐rTRIM21 or SFB‐rTRIM21 ligase‐dead (LD) mutant were transfected with HA‐Ub. MG132 (10 µm) was added to the cells 6 h before they were harvested with guanidine‐HCl‐containing buffer. Immunoprecipitation was performed with an anti‐CD47 antibody. G) U251 cells stably expressing TRIM21 shRNA or control shRNA were reconstituted with WT SFB‐rTRIM21 or the SFB‐rTRIM21 LD mutant. H) CD47‐depleted U251 cells with reconstituted expression of WT Flag‐rCD47 or the indicated Flag‐tagged mutants were stimulated with or without EGF (100 ng mL^−1^) for 24 h. The results represent the means ± SD; ANOVA two‐way test. Different letters indicate significant differences (*p* < 0.05). I) CD47‐depleted U251 cells with reconstituted expression of WT Flag‐rCD47 or Flag‐rCD47 K99/102R mutant were co‐transfected with His‐tagged TRIM21 and HA‐Ub. MG132 (10 µm) was added to the cells 6 h before they were harvested with guanidine‐HCl‐containing buffer. Immunoprecipitation was performed with an anti‐Flag antibody. J) U251 cells were reconstituted with WT Flag‐CD47 or Flag‐CD47 K99/102R mutant and then treated with CHX (100 µg mL^−1^) for the indicated periods of time. Quantification of relative Flag (CD47) protein levels is shown (right panel).

To delineate the mechanism underlying CD47 Y288 phosphorylation‐inhibited CD47 polyubiquitylation, we performed mass spectrum analyses of immunoprecipitated CD47 and found that the ubiquitin E3 ligase TRIM21 is a CD47‐associated protein (Figure S5A, Supporting Information). This association was validated by coimmunoprecipitation analyses (**Figure** **5**A). Notably, EGF treatment for 1 h (Figure 5A) or expression of activated c‐Src CA (Figure S5B, Supporting Information), which enhanced CD47 expression, reduced the binding of CD47 to TRIM21. Compared to its WT counterpart, CD47 Y288F exhibited consistent binding to TRIM21, which was resistant to EGF treatment‐ (Figure 5B) or activated c‐Src CA expression‐induced (Figure S5C, Supporting Information) disruption of the TRIM21‐CD47 complex. These results indicated that EGF‐induced and c‐Src‐mediated CD47 Y288 phosphorylation inhibits the association between TRIM21 and CD47.

We next depleted TRIM21 by expressing its shRNAs in U251 cells and showed that TRIM21 depletion reduced CD47 polyubiquitylation (Figure 5C) with a correspondingly increased half‐life (Figure S5D, Supporting Information) and expression (Figure 5D) of CD47. In contrast, overexpression of S protein‐FLAG‐streptavidin binding peptide (SFB)‐tagged TRIM21 enhanced CD47 polyubiquitylation (Figure S5E, Supporting Information) and reduced the half‐life (Figure S5F, Supporting Information) and expression (Figure 5E) of CD47. Compared to WT TRIM21 expression, reconstituted expression of the TRIM21 E3 ligase‐dead mutant (LD) in endogenous TRIM21‐depleted U251 cells reduced CD47 polyubiquitylation (Figure 5F) and elevated CD47 levels (Figure 5G). These results indicated that EGF‐induced c‐Src activation results in the dissociation of TRIM21 from CD47, thereby inhibiting TRIM21‐mediated CD47 polyubiquitylation and degradation.

To identify the polyubiquitylation lysine (K) in CD47 mediated by TRIM21, we analyzed the CD47 protein sequence with a polyubiquitylation prediction software (www.ubpred.org). Expression of the potential polyubiquitylation residue mutants showed that CD47 K99R and CD47 K102R increased their basal expression levels, and combined CD47 K99/102R mutations further enhanced CD47 expression, which was resistant to EGF‐ (Figure 5H) and c‐Src CA‐induced (Figure S5G, Supporting Information) upregulation. In addition, CD47 K99/102R mutations abolished TRIM21‐mediated CD47 polyubiquitylation (Figure 5I) and extended CD47 half‐life (Figure 5J). These results indicated that EGF‐induced c‐Src activation inhibits TRIM21‐mediated CD47 polyubiquitylation and degradation.

### 2.6 EGFR Activation‐Induced and c‐Src‐Mediated CD47 Phosphorylation and Stabilization Promote Immune Evasion of Tumor Cells and Brain Tumor Growth

**Figure 6:**
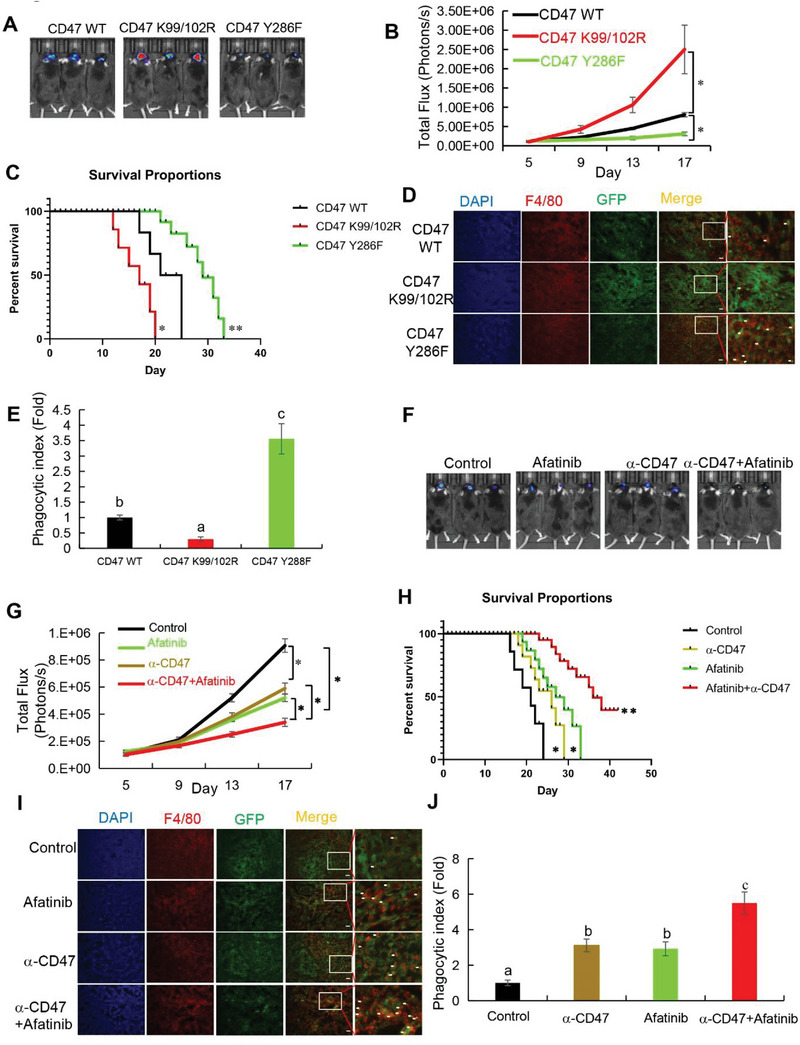
EGFR activation‐induced and c‐Src‐mediated CD47 phosphorylation and stabilization promote immune evasion of tumor cells and brain tumor growth. A) A total of 1 × 10^5^ mouse CT‐2A/Luc‐GFP cells with or without knockout of CD47 or knock‐in of CD47 Y286F or K99/102R mutant were intracranially injected into C57BL/6 mice. After 15 days, the mice were euthanized and examined for tumor growth. Representative tumor growth was shown in vivo by bioluminescence imaging using IVIS 100. B) A bioluminescence imaging analysis of tumor burden was performed on the indicated days. C) The mouse survival times were recorded and visualized using Kaplan–Meier survival curves. D) Immunofluorescent staining of the mouse GBM specimens was performed with the indicated antibodies. The macrophages that engulfed the cancer cells were indicated with arrows. Scale bar, 50 µm. E) Tumor macrophage phagocytosis was estimated by quantification of the phagocytic index (*n* = 4). The results represent the means ± SD; ANOVA two‐way test. Different letters indicate significant differences (*p* < 0.05). F) A total of 1 × 10^5^ CT‐2A/Luc‐GFP cells were intracranially injected into syngeneic C57BL/6 mice. After 15 days, the mice were euthanized and examined for tumor growth. Representative tumor growth was shown in vivo by bioluminescence imaging using IVIS 100. G) A bioluminescence imaging analysis of tumor burden was performed on the indicated days. H) The mouse survival times were recorded and visualized using Kaplan–Meier survival curves. I) Immunofluorescent staining of the mouse GBM specimens was performed with the indicated antibodies. The macrophages that engulfed the cancer cells were indicated with arrows. Scale bar, 50 µm. J) Tumor macrophage phagocytosis was estimated by quantification of the phagocytic index (*n* = 4). The results represent the means ± SD; ANOVA two‐way test. Different letters indicate significant differences (*p* < 0.05).

We next examined the role of EGF‐induced and c‐Src‐upregulated CD47 expression in tumorigenesis and tumor immune evasion in mice. We intracranially injected luciferase‐expressing CT‐2A mouse glioma cells with or without knock‐in expression of CD47 Y286F (Figure S6A, Supporting Information) or CD47 K99/102R (Figure S6B, Supporting Information). Expression of CD47 Y286F inhibited tumor growth (**Figure** **6**A,B), prolonged mouse survival time (Figure 6C), and reduced CD47 expression in tumor tissues, as detected by IHC analysis (Figure S6C, Supporting Information). In contrast, CD47 K99/102R expression, which increased CD47 expression in tumors (Figure S6C, Supporting Information), accelerated tumor growth (Figure 6A,B) and shortened mouse survival time (Figure 6C). Notably, CD47 Y286F and CD47 K99/102R expression increased and decreased phagocytosis of the GFP‐expressing tumor cells by infiltrated tumor‐associated macrophages (TAMs), respectively, as detected by immunofluorescence staining of F4/80 a macrophage marker (Figure 6D,E and Figure S6D, Supporting Information, left table). These results indicated that EGFR activation‐induced and c‐Src‐mediated CD47 Y286 phosphorylation and subsequent CD47 stabilization inhibit macrophage‐mediated tumor cell phagocytosis and promote tumor growth.

To examine whether inhibition of CD47 can sensitize the inhibitory effect of EGFR inhibitors on tumor growth, we treated the mice bearing the brain tumor derived from CT‐2A glioma cells with EGFR inhibitor afatinib or an anti‐CD47 antibody alone or with a combination of both reagents. Afatinib or anti‐CD47 antibody treatment alone inhibited tumor growth (Figure 6F,G) and prolonged mouse survival (Figure 6H). Notably, the combined treatment of afatinib and the anti‐CD47 antibody resulted in an additive effect on tumor growth inhibition (Figure 6F,G) and mouse survival prolongation (Figure 6H). IHC analyses of tumor tissues showed that afatinib, but not the anti‐CD47 antibody, treatment reduced the levels of EGFR p1068, c‐Src pY416, and CD47 expression (Figure S6E, Supporting Information). Analyses of phagocytosis of the tumor cells by macrophage revealed that afatinib and the anti‐CD47 antibody treatment each alone enhanced phagocytosis and that a stronger effect on phagocytosis was observed upon combined treatment with both reagents (Figure 6I,J and Figure S6D, Supporting Information, right table). These results suggested that CD47‐SIRPα blockade improves EGFR‐targeted cancer therapy.

### 2.7 CD47 Levels Positively Correlate with the Levels of Activities of EGFR and c‐Src in Human GBM and a Poor Prognosis in GBM Patients

**Figure 7:**
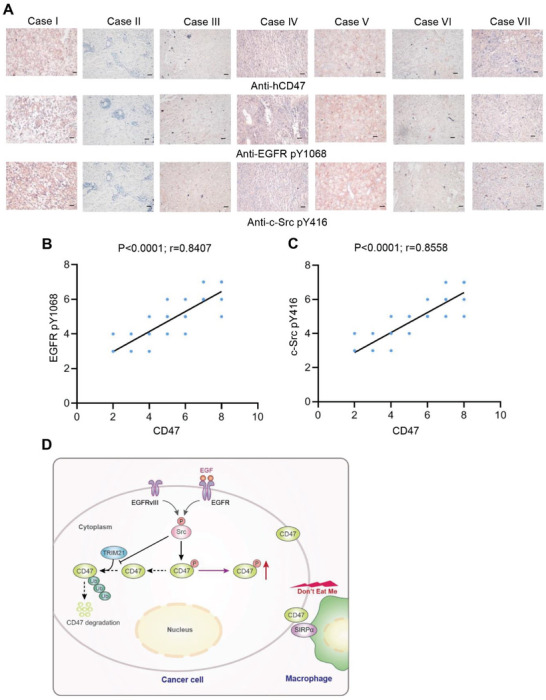
Activation of the EGFR/c‐Src pathway correlates with CD47 expression in human GBM specimens. A) IHC staining of 25 human GBM specimens was performed with the indicated antibodies. Representative images from the staining of seven different specimens are shown. Scale bar, 100 µm. B,C) The IHC stains were scored, and correlation analyses were performed. The Pearson correlation test was used. Note that the scores of some samples overlap. D) A schematic of c‐Src‐regulated CD47 phosphorylation and expression.

To determine the clinical relevance of EGFR and c‐Src activation‐regulated CD47 expression, we analyzed 25 human GBM specimens and showed that the levels of CD47 expression positively correlated with EGFR pY1068 and c‐Src pY416 (**Figure** **7**A–C). These results suggested that EGFR and c‐Src activation‐induced CD47 expression is associated with aggravation of human GBM.

## 3 Discussion

Tumor cells often overexpress immune checkpoint proteins, including CD47, for immune evasion.^[^ ^2^ ^]^ Activation of RTKs in GBM is a crucial driver event in GBM development.^[^ ^8b^ ^]^ However, whether these oncogenic signalings regulate CD47 expression is not known. We demonstrated here that CD47 expression levels correlated with the grades of human gliomas and with a poor prognosis in GBM patients. Mechanistically, amplification or mutation of EGFR, which are the dominant receptor tyrosine kinase lesions in GBM and occur in 57% of tumors,^[^ ^10^ ^]^ induced CD47 expression independent of its transcriptional and translational regulation. EGFR activation increased the binding of c‐Src to CD47, and EGFR‐activated c‐Src phosphorylated CD47 at Y288. This phosphorylation inhibited the interaction between TRIM21 and CD47, thereby abrogating TRIM21‐mediated CD47 K99/102 polyubiquitylation and CD47 degradation. Knock‐in expression of CD47 Y288F reduced CD47 expression, increased phagocytosis of the tumor cells by macrophage, and reduced tumor growth. In contrast, expression of CD47 K99/102R increased CD47 expression with correspondingly decreased macrophage phagocytosis of the tumor cells and promoted tumor growth. Importantly, CD47‐SIRPα blockade with an anti‐CD47 antibody treatment significantly enhanced EGFR‐targeted cancer therapy and elicited much greater tumor growth inhibition than either treatment alone (Figure 7D).

Posttranslational modifications of proteins play critical roles in the regulation of protein stability,^[^ ^11^ ^]^ and under certain circumstances, they can also modulate protein functions without altering their expression levels.^[^ ^11^, ^12^ ^]^ Pyroglutamate modification at the SIRPα‐ binding site of CD47 by glutaminyl‐peptide cyclotransferase‐like (QPCTL), which did not affect cell surface CD47 levels, promotes the binding of CD47 to SIRPα. Interference with QPCTL expression enhanced antibody‐dependent tumor cell phagocytosis and increased neutrophil‐mediated tumor cell killing.^[^ ^13^ ^]^ Thus, CD47‐SIRPα immune checkpoint can be modulated by governing CD47 functions through signaling context‐dependent regulation.

SIRPα is expressed on the surface of natural killer (NK) cells and myeloid cells including macrophages, neutrophils, and subsets of dendritic cells.^[^ ^3^, ^14^ ^]^ Elevated expression of CD47 protected tumor cells against SIRPα‐expressing primary NK cell killing while disruption of CD47‐SIRPα augments NK cell antitumor responses.^[^ ^15^ ^]^ In addition, blocking the CD47‐SIRP interaction may potentiate neutrophil‐mediated antibody‐dependent cellular cytotoxicity (ADCC) toward cancer cells.^[^ ^16^ ^]^ CD47 blockade in combination with temozolomide treatment was also shown to promote glioma cell phagocytosis by APCs and subsequent enhancement of antigen cross‐presentation and activation of APCs for more efficient T cell priming.^[^ ^17^ ^]^ These reports together our finding that CD47‐SIRPα blockade significantly enhanced EGFR‐targeted cancer therapy by substantiated macrophage phagocytosis of the tumor cells reveal the great potential to enhance specific or combined anti‐tumor immune responses by eliminating CD47‐mediated inhibition of various types of immune cells.

Treatments that target immune checkpoints mediated by CD47 and SIRPα are currently under clinical trials for treating human cancer.^[^ ^18^ ^]^ However, how to stratify patients to maximize treatment efficacy remains under investigation. For non‐small lung cancer cells, CD47 expression was upregulated in the cancer cells' resistance to EGFR inhibitor treatment, and CD47 blockade increased the clearance of the resistant cells by phagocytes.^[^ ^19^ ^]^ Given that receptor tyrosine kinase‐targeted therapies are prevalent in treating many types of cancer, our findings elucidate a novel mechanism underlying CD47 expression in RTK‐activated tumor cells and underscore the synergetic tumor inhibition effect elicited by combined treatment with EGFR inhibitors and CD47‐SIRPα blockade. In addition, the association of EGFR, c‐Src activation‐induced CD47 Y288 phosphorylation and CD47 expression with aggravation of human GBM highlights the significance of this signaling axis in GBM tumor evasion and tumor development and accentuates the need to improve the current cancer therapy and eliminate drug resistance with a combination of CD47‐SIRPα blockade with RTK‐targeted treatment.

## 4 Experimental Section

### Materials

A rabbit polyclonal antibody that recognizes CD47 (pY288) was generated by Bioworld (Nanjing, China). The synthesized peptide GLVpYMKFVASNQC representing amino acids from 285 to 297 of CD47 (CD47 pY288) was injected into rabbits. After that, the serum was collected and purified by using an affinity column conjugated to synthesized non‐phosphorylated CD47 peptide (GLVYMKFVASNQC) to exclude the antibodies recognizing non‐phosphorylated CD47, followed by an affinity column conjugated to phosphorylated CD47 pY288 peptide to bind and purify the CD47 pY288 antibody. A working concentration of 1 µg mL^−1^ was used for immunoblotting.

Normal rabbit immunoglobulin (sc‐2027) and a polyclonal antibody against mouse TRIM21 (sc‐21367, M‐20, 1:1000 for immunoblotting) were purchased from Santa Cruz Biotechnology (Santa Cruz, CA). Rabbit monoclonal antibodies that recognize human EGFR (4267, 1:1000 for immunoblotting), EGFR (pY1068, 3777, 1;1000 for immunoblotting, 1:400 for IHC), c‐Src (2109, 1;1000 for immunoblotting, 1:50 for immunoprecipitation), AKT (pT308, 4056, 1:1000 for immunoblotting), AKT (9272, 1:1000 for immunoblotting), ERK1/2 (pT202/pY204, 9101, 1:1000 for immunoblotting), HA (3724, 1;1000 for immunoblotting, 1:50 for immunoprecipitation), and ERK1/2 (9102, 1:1000 for immunoblotting) were purchased from Cell Signaling Technology (Danvers, MA). Mouse monoclonal antibodies for FLAG (F3165, M2, 1:5000 for immunoblotting, 1:1000 for immunoprecipitation) and polyclonal antibodies for c‐Src (pY416, SAB5701768, 1:1000 for immunoblotting, 1:100 for IHC) were purchased from Sigma (St. Louis, MO). Polyclonal antibodies against human TRIM21 (ab91423, 1:2000 for immunoblotting) and mouse F4/80 (300 421, 1:50 for immunofluorescence) were obtained from Abcam (Cambridge, MA). A polyclonal antibody against tubulin (10 068, 1:5000 for immunoblotting) was purchased from Proteintech (Wuhan, China). Polyclonal antibodies against human CD47 (AF4670, 1 µg mL^−1^ for immunoblotting) and mouse CD47 (AF1866, 1 µg mL^−1^ for immunoblotting) were obtained from R&D Systems (Minneapolis, MN). A monoclonal antibody against human CD47 (B6H12, 1:100 for IHC, flow cytometry, and immunoprecipitation) was obtained from Thermo Fisher Scientific (Waltham, MA). The rat anti‐mouse CD47 antibody (BE0270) for the animal study was purchased from Bio X Cell (Lebanon, NH). Human recombinant EGF (01‐407) was obtained from EMD Millipore (Billerica, MA). Hygromycin (400 053), puromycin (540 222), and G418 (345 810) were purchased from EMD Biosciences (San Diego, CA). Actinomycin D, cycloheximide (CHX), afatinib, AZD9291, AZD3759, Su6656, U0126, MK2206, and MG132 were purchased from MedChemExpress (NJ, USA). Active GST‐c‐Src (S19‐18G) was obtained from Signalchem (Richmond, BC, Canada). Lipofectamine 2000 transfection reagent was obtained from Thermo Fisher Scientific (Waltham, MA).

### Cell Culture and Transfection

NHA and GBM cells, including U251, U87, A172, LN229, LN18, U373, and T98G cells, were obtained from ATCC and were routinely tested for mycoplasma. The U87 and U251 cell lines in the experiments were authenticated using short tandem repeat profiling at The University of Texas MD Anderson Cancer Center. HEK293T cells, EGFR or EGFR/vIII‐overexpressing HEK293T (HEK293T/EGFR or HEK293T/EGFRvIII) cells, NHA cells, CT‐2A cells, CT‐2A cells overexpressing firefly luciferase fused with GFP (CT‐2A/Luc‐GFP), and human GBM cells, including EGFR‐overexpressing U87 (U87/EGFR) and EGFRvIII‐overexpressing U87 (U87/EGFRvIII) cells, were maintained in Dulbecco's modified Eagle's medium (DMEM) supplemented with 10% bovine calf serum (HyClone, Logan, UT). GL261 cells were maintained in 50/50 DMEM/F‐12 supplemented with 10% bovine calf serum. Human primary GBM cells (GSC7‐11 and GSC6‐27) were maintained in 50/50 DMEM/F‐12 supplemented with B27 (Thermo Fisher Scientific, Waltham, MA), EGF (10 ng mL^−1^), and basic fibroblast growth factor (Thermo Fisher Scientific, 10 ng mL^−1^). Cells were plated at a density of 4 × 10^5^ per 60‐mm dish or 1 × 10^5^ per well of a 6‐well plate 18 h before transfection. The transfection procedure was performed as previously described.^[^ ^20^ ^]^

### DNA Constructs and Mutagenesis

PCR‐amplified human CD47 (hCD47), c‐Src, and mouse CD47 (mCD47) were cloned into pcDNA3.1/hygro(+) (Thermo Fisher Scientific, Waltham, MA), pcDNA3.1/hygro(+)‐Flag (Thermo Fisher Scientific, Waltham, MA), pCDH‐CMV‐MCS‐EF1‐Puro‐SFB(Addgene, Cambridge, MA), or pCold/his (Takara, Shiga, Japan) vectors. pcDNA3.1/hygro(+)‐Flag hCD47 Y288F, hCD47 K74R, hCD47 K85R, hCD47 K99R, hCD47 K102R, hCD47 K99/102R, hCD47 K290R, mCD47 Y286F, mCD47 K99/102R, human rCD47‐Flag (resistance to shRNA), pCold/his‐hCD47 Y288F, and pcDNA3.1/hygro(+)‐c‐Src Y530F (constitutively active c‐Src; c‐Src CA) were generated using the QuikChange site‐directed mutagenesis kit (Stratagene, La Jolla, CA). pCDH‐CMV‐MCS‐EF1‐Puro‐SFB TRIM21 and pCDH‐CMV‐MCS‐EF1‐Puro‐SFB LD (C16A, C31A, and H33W) were created in the previous study.^[^ ^21^ ^]^

The following pGIPZ shRNAs were used: control shRNA oligonucleotide, GCTTCTAACACCGGAGGTCTT; c‐Src shRNA#1 oligonucleotide, CGAGGAGGTGTACTTTGAGAA; c‐Src shRNA#2 oligonucleotide, GAGAAGGGCTACAAGATGGAT; TRIM21 shRNA#1 oligonucleotide, AGTATCAGCCACGGATTGG; TRIM21 shRNA#2 oligonucleotide, TCCAGAGTGAAAGTGCTGG; and CD47 shRNA oligonucleotide, GCCTTGGTTTAATTGTGACTT.

### Reverse Transcription and PCR Analysis

Total RNA isolation, reverse transcription (RT), and real‐time PCR were performed as described previously.^[^ ^22^ ^]^ The primers used for quantitative real‐time PCR shown as followed: human CD47, 5′‐GAAGTGGGTATTGTGGTGGG‐3′ (forward) and 5′‐AGGCTCTGGTGGCTGCTCTAA‐3′ (reverse); mouse CD47, 5′‐GAGTTATCCAGAGAAGGCAAAAC‐3′ (forward) and 5′‐CGTATGGCTGGATTTATATTTGAG‐3′ (reverse); and β‐actin, 5′‐ATGGATGACGATATCGCTGCGC‐3′ (forward) and 5′‐GCAGCACAGGGTGCTCCTCA‐3′ (reverse).

### Purification of Recombinant Proteins

His‐hCD47 WT and His‐hCD47 Y288F were expressed in BL21 (DE3) cells and then purified by His‐NTA resin (GE Healthcare, Pittsburgh, PA).^[^ ^23^ ^]^ Briefly, pCold His‐CD47 WT and pCold hCD47 Y288F were transformed into BL21/DE3 bacteria. Transformants were screened and selected to inoculate 10 mL cultures of LB/ampicillin, which were grown overnight at 37 °C to stationary phase. 2 mL of precultured bacterial medium was then used to inoculate 50 mL of LB/ampicillin. The cultures were grown at 37 °C to an OD600 of ≈0.4–0.6 before the addition of 0.5 mm IPTG at 16 °C for 24 h. After that, cell pellets were collected by centrifuging at 5000 r.p.m. for 5 min at 4 °C, resuspended in 10 mL Bugbuster protein extraction reagent (Sigma‒Aldrich) with the addition of 20 µL protease cocktail inhibitor (Roche), and incubated at room temperature for 20 min before centrifuging at 10 000 r.p.m. for 10 min at 4 °C. Cleared lysates were then bound to Ni‐NTA His Bind Resin for 12 h with rolling at 4 °C. The beads were washed with extraction buffer for 5 min and rolled at 4 °C three times. Then, the beads were collected and eluted with extraction buffer (pH 7.5) plus 500 mm imidazole for 1 h with rolling at 4 °C. The purified proteins were then dialyzed at 20 mm Tris‐HCl pH 7.5, 50 mm NaCl, 10% glycerol, and 1 mm dithiothreitol at 4 °C overnight.

### In Vitro Kinase Assay

GST‐c‐Src (500 ng) was incubated with His‐CD47 or His CD47 Y288F (200 ng) in 25 µL of kinase buffer (50 mm Tris‐HCl (pH 7.5), 100 mm KCl, 50 mm MgCl_2_, 1 mm Na_3_VO_4_, 1 mm DTT, 5% glycerol and 0.5 mm ATP) at 25 °C for 1 h. The reaction was terminated by adding SDS‒PAGE loading buffer and heating at 100 °C for 5 min.^[^ ^24^ ^]^ The reaction mixture was then subjected to SDS‒PAGE and immunoblotting.

### Immunoprecipitation and Immunoblotting Analysis

Proteins were extracted from cultured cells using a modified buffer, followed by immunoprecipitation and immunoblotting with the corresponding antibodies.^[^ ^25^ ^]^ Each experiment was repeated at least three times.

### Mass Spectrometry Analysis

An in vitro c‐Src‐phosphorylated purified CD47 was digested in gel in 50 mm ammonium bicarbonate buffer containing RapiGest (Waters Corp., Milford, MA) overnight at 37 °C with 200 ng of sequencing‐grade modified trypsin (Promega, Madison, WI). The digest was analyzed by LC‐MS/MS on an Orbitrap‐Elite mass spectrometer (Thermo Fisher Scientific, Waltham, MA). Proteins were identified by searching for the fragment spectra in the Swiss‐Prot protein database (EBI) using the Mascot search engine (version 2.3; Matrix Science, London, UK) and SEQUEST v.1.27 (University of Washington, Seattle, WA) via the Proteome Discoverer software program (version 1.4; Thermo Fisher Scientific, Waltham, MA). Phosphopeptide matches were analyzed using the phosphoRS algorithm implemented in Proteome Discoverer and manually curated.^[^ ^26^ ^]^

### In Vivo Ubiquitylation Assay

Cells were transfected with the indicated plasmids for 48 h and lysed using denaturing buffer (6 m guanidine‐HCl (pH 8), 0.1 m Na_2_HPO_4_/NaH_2_PO_4_, and 10 mm imidazole) containing 5 mm *N*‐ethylmaleimide to prevent deubiquitylation.^[^ ^21^, ^27^ ^]^ The cell lysates were immunoprecipitated using the indicated antibodies, washed, and subjected to immunoblotting analysis.

### Animal Experiments

Different types (wild type, CD47 Y286F, and CD47 K99/102R) of 1 × 10^5^ CT‐2A/Luc‐GFP cells (in 5 µL of DMEM/F12 per mouse) were intracranially injected into 4‐week‐old male C57BL/6 mice. Each group of mice was divided into two subgroups. Each subgroup contained seven mice and was used to monitor tumor growth and perform IHC analyses. The mice were euthanized 15 days after the CT‐2A/Luc‐GFP cells were injected. The brain of each mouse was harvested and fixed in 4% formaldehyde; half of the brain was embedded in paraffin for IHC staining, and the surplus brain was stored at −80 °C for further IF staining. Tumor formation and phenotype were determined by bioluminescence imaging and histological analysis of hematoxylin and eosin‐stained sections.^[^ ^28^ ^]^

The other subgroups, which consisted of nine mice, were monitored for survival. The results were analyzed using STATISTICA software and represented by Kaplan–Meier plots. Humane endpoints included weight loss of 20–25%, weakness that prevented them from obtaining food or water, loss of appetite (anorexia for 24 h), moribund state, and an inability to participate in normal activities because of tumor growth. All mice were euthanized under anesthesia after two or more of these humane endpoints were observed.

For treatment with afatinib and antibody, 100 mg of rat anti‐CD47 antibody (Bio X Cell) or rat IgG (Bio X Cell) as control was injected intratumorally on days 4, 7, 10, 13, and 16 after CT‐2A/Luc‐GFP cell inoculation and afatinib (120 mg kg^−1^) was injected intraperitoneally on days 3, 5, 7, 9, 11, 13, and 15.

All of the mice were housed in the Wenzhou Medical University Animal Center (Wenzhou, China). All animal experiments were performed according to the guidelines and regulations of the Zhejiang Medical Laboratory Animal Management Committee.

### Bioluminescence Imaging and Analysis

The mice were intraperitoneally injected with 150 mg kg^−1^ d‐luciferin in PBS. 10 min after injection, bioluminescence imaging was conducted using a charge‐coupled device camera (IVIS 200, Xenogen; exposure time of 30 s, binning of 8, a field of view of 15 cm, f/stop of 1, and no filter). Mice were anesthetized with isoflurane (2% vaporized in O_2_). For analysis, the total photon flux (photons per second) was measured from a fixed region of interest using Living Image software (Xenogen). Bioluminescent signals within the fixed region of interest were normalized to the background luminescence and were obtained over the same region of interest from animals that had not been injected with d‐luciferin.^[^ ^29^ ^]^

### IHC Analysis and Scoring

The human GBM samples and clinical information were from TCGA (https://www.cbioportal.org/). This study was approved by the Ethics Committee of Wenzhou Medical University (China), and written informed consent was obtained from all patients. Tissue sections from 30 paraffin‐embedded human GBM specimens were stained with antibodies against CD47, EGFR (pY1068), c‐Src (pY416), or nonspecific IgG as a negative control. The tissue sections were quantitatively scored according to the percentage of positive cells and staining intensity. The following proportion scores were assigned:^[^ ^30^ ^]^ 0 if 0% of the tumor cells showed positive staining, 0.1–1 if 0.1% to 1% of cells were stained, 1.1–2 if 1.1% to 10% of cells were stained, 2.1–3 if 11% to 30% of cells were stained, 3.1–4 if 31% to 70% of cells were stained, and 4.1–5 if 71% to 100% of cells were stained. The intensity of staining was rated on a scale of 0 to 3: 0, negative; 1, weak; 2, moderate; and 3, strong. The proportion and intensity scores were then combined to obtain a total score (range, 0–8). All GBM patients from whom the tumor samples were obtained had undergone standard radiotherapy after surgery followed by treatment with an alkylating agent (temozolomide in most cases). Consent for the collection of patient specimens, the use of human brain tumor specimens, and the database were approved by the committee for ethical review of research at the First Affiliated Hospital of Wenzhou Medical University in Wenzhou, China.

### In Vivo Phagocytosis Assay

For immunofluorescence staining, orthotopic GFP‐expressing CT‐2A‐derived tumor tissues were fixed in 4% paraformaldehyde and embedded with OCT compound at a 5 µm thickness. Slides were blocked in PBS containing 10% normal goat serum and 0.3% Triton X‐100 for 1 h at room temperature and then incubated with a primary antibody cocktail of rabbit anti‐mouse F4/80 (Abcam, 1:100) with 1% bovine serum albumin. After washing three times with 1% BSA in PBS, the slides were incubated with a Cy3‐conjugated goat anti‐rabbit IgG (Beyotime, Beijing, China, 1:200 diluted in PBS, which included 10% goat serum) as a secondary antibody. Cell nuclei were stained using an antifade solution containing DAPI (Beyotime). Then, the phagocytosis of tumor cells was determined under fluorescence microscopy (Nikon). The phagocytic index was calculated by the following formula: phagocytic index = (number of engulfed cells/total number of macrophages containing engulfed cells) × (total number of macrophages containing engulfed cells/total number of counted macrophages) × 100.

### Genomic Editing

Genomic mutations in CT‐2A/Luc‐GFP cells were created using the CRISPR/Cas9 system as described previously.^[^ ^31^ ^]^ sgRNAs were designed to target the genomic areas adjacent to the mouse CD47 mutation sites (Y286F sgRNA target sequence: ATAGCTCTAGCAGAACTACT; CD47 K99/102R sgRNA target sequence: TCAGTCTCAGACTTAATCAA). The annealed guide oligos containing overhangs were inserted into the PX458 vector (Addgene, Cambridge, MA) digested by the BbsI restriction enzyme. In a 24‐well plate, U87 cells at 60% confluence were co‐transfected with a single‐stranded donor oligonucleotide (ssODN) (20 pmoles) that was used as a template to introduce the mutations for generating the mCD47 Y286F sequence: TATTTCATTTGTCTCTACAGCATGTGAGCCAGTGCACGGCCCCCTTTTGATTTCAGGTTTGGGGATCATAGCTTTGGCTGAGCTACTTGGATTAGTTTTTATGAAGTTTGTCGGTAAGTTAATCTTACTTTTTAAGCCTCTGCAAAGAATTTCAGATACTTAGAAAATAGATTTTGTGCATCCTTTGAGCTGGGGGTAGG or the mCD47 K99/102R sequence (TTTCATCTATGATGGAAATAAAAATAGCACTACTACAGATCAAAACTTTACCAGTGCAAAAATCTCAGTCAGCGACCTCATCAATGGCATTGCCTCTTTGAGAATGGATAGGCGCGATGCCATGGTGGGAAACTACACTTGCGAAGTGACAGAGTTATCCAGAGAAGGCAAAACAGTTATAGAGCTGAAAAACCGCACGG) along with a vector (0.5 µg) able to coexpress a sgRNA targeting the CD47 gene and a wild‐type hSpCas9 tagged with GFP. 24 h after transfection, cells were trypsinized and diluted in the medium for single‐cell seeding into a 96‐well plate, and GFP‐positive cells were marked and subjected to genomic DNA extraction. Genotyping was performed by sequencing the PCR products amplified using primers (mCD47 Y286F forward: AATGGTAGTACTGTTTGCCTAG; mCD47 Y286F reverse: GGTACTTTGACTTCACTTTGCT; mCD47 K99/102R forward: GTTCAGCTCAACTACTGTTTAG; mCD47 K99/102R reverse: CGTGCGGTTTTTCAGCTCTAT) spanning the mutation area.

### Statistical Analysis

All quantitative data are presented as the mean ± SD of at least three independent experiments. A two‐group comparison was conducted using a two‐sided, two‐sample Student's *t*‐test. Simultaneous comparison of more than two groups was conducted using one‐way ANOVA (SPSS statistical package, version 12; SPSS Inc.). Values of *p* < 0.05 were considered statistically significant.

## Conflict of Interest

The authors declare no conflict of interest.

## Author Contributions

L.D. and Z.S. contributed equally to this work. Z.L. and J.L. conceptualized the studies and wrote the manuscript. L.D. and J.‐H.L. performed most of the experiments and data analysis. Z.S., S.W., F.X., and S.B.L. performed experiments and collected the gliomas specimen. D.X., X.J., and X.Q. contributed methodology and experimental resources. Y.M. helped design experiments and provided technical assistance. All authors have read and approved the final version of the manuscript.

## Supporting information

Supporting information

## Data Availability

The data that support the findings of this study are available in the supplementary material of this article.
