# Supplementary material for: EGFR‐Induced and c‐Src‐Mediated CD47 Phosphorylation Inhibits TRIM21‐Dependent Polyubiquitylation and Degradation of CD47 to Promote Tumor Immune Evasion
Source: Adv Sci (Weinh). 2023 Aug 4;10(27):2206380. doi: 10.1002/advs.202206380 (PMC10520678; doi:10.1002/advs.202206380)
Supplement: Supplementary file 1 — Supporting information [file ADVS-10-2206380-s001.pdf]

ADVANCED SCIENCE
Open Access

Supporting Information

for *Adv. Sci.*, DOI 10.1002/advs.202206380

EGFR-Induced and c-Src-Mediated CD47 Phosphorylation Inhibits TRIM21-Dependent Polyubiquitylation and Degradation of CD47 to Promote Tumor Immune Evasion

Linyong Du, Zhipeng Su, Silu Wang, Ying Meng, Fei Xiao, Daqian Xu, Xinjian Li, Xu Qian, Su Bin Lee, Jong-Ho Lee, Zhimin Lu* and Jianxin Lyu**

Supplementary material

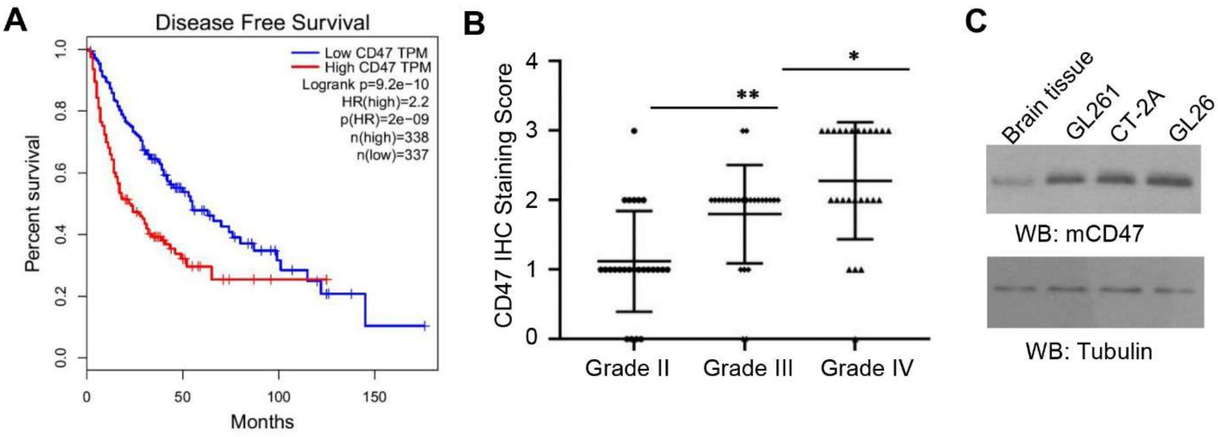

Figure S1. CD47 expression is upregulated in GBM tissues.

(A) Kaplan–Meier 15-year disease-free survival analysis comparing CD47 high- and low-expressing patients in the TCGA GBM cohort. The CD47 high and low groups were separated by the median expression. Significance was determined with the log-rank test. $P=9.2\times10^{-10}$; HR, 2.2.

(B) Quantification of CD47 expression in human glioma specimens of different grades (II-IV).

(C) The protein expression levels of CD47 in mouse normal brain tissue and the indicated mouse glioma cells were determined by immunoblotting analyses.

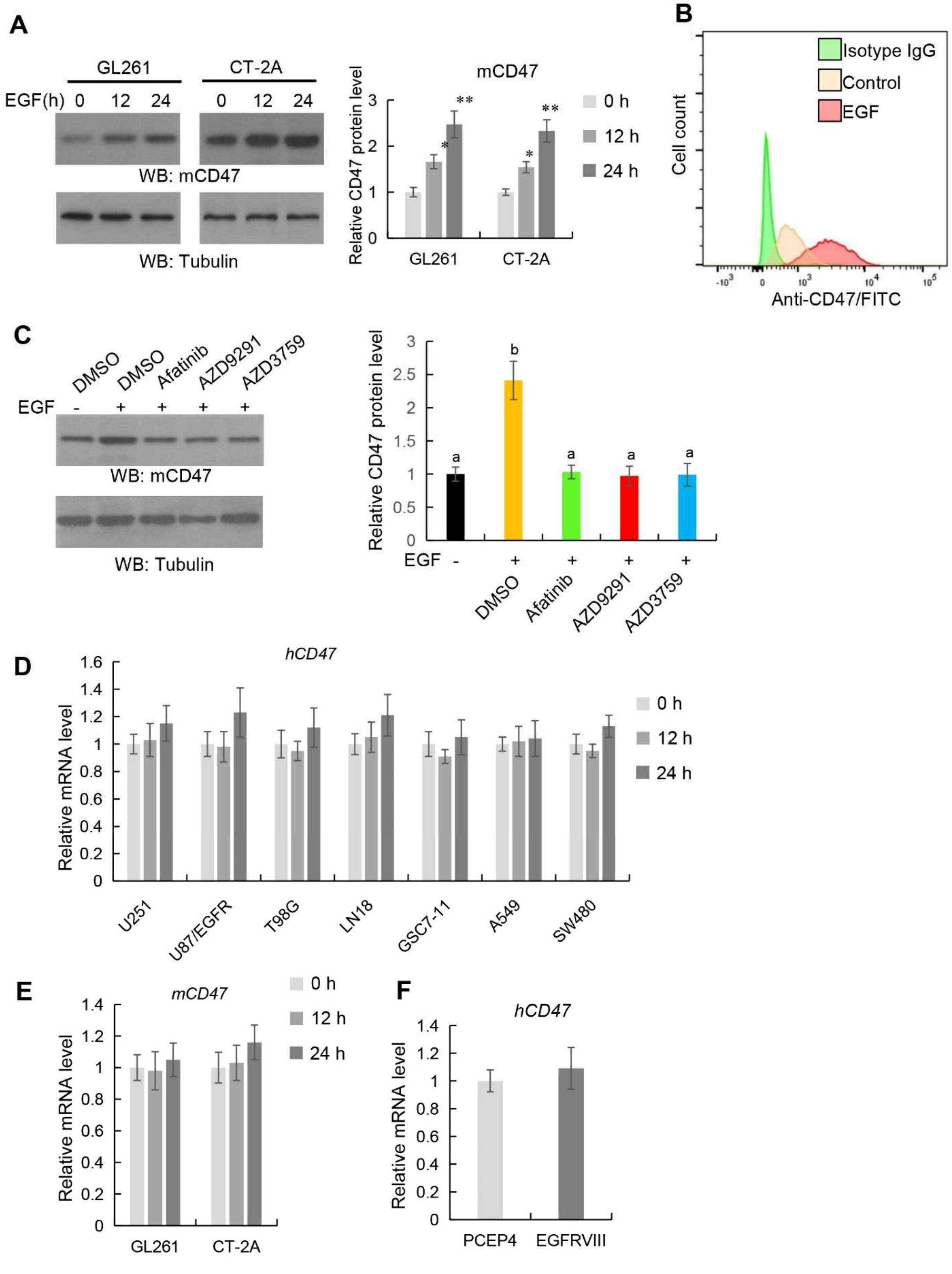

Figure S2. EGFR activation induces CD47 protein upregulation.

(A) The indicated murine glioma cells were serum-starved for 12 h and then stimulated with EGF (100 ng/ml) for the indicated periods of time. Immunoblot analyses were performed with the indicated antibodies (left panel). Quantification of relative CD47 protein levels is shown (right panel).

(B) U251 cells were treated with or without EGF (100 ng/ml) for 24 h. Cell surface analysis of CD47 protein was performed using a flow cytometer.

(C) Serum-starved CT-2A cells were stimulated with EGF (100 ng/ml) for 24 h in the presence or absence of the indicated EGFR inhibitors. Immunoblot analyses were performed with the indicated antibodies (left panel). Quantification of relative CD47 protein levels is shown (right panel).

(D and E) The indicated human tumor cells (D) and murine glioma cells (E) were serum-starved for 12 h and then stimulated with EGF (100 ng/ml) for the indicated periods of time. Real-time PCR analyses were performed.

(F) U87 cells were stably transfected with plasmids expressing control vector or EGFRvIII. Real-time PCR analyses were performed.

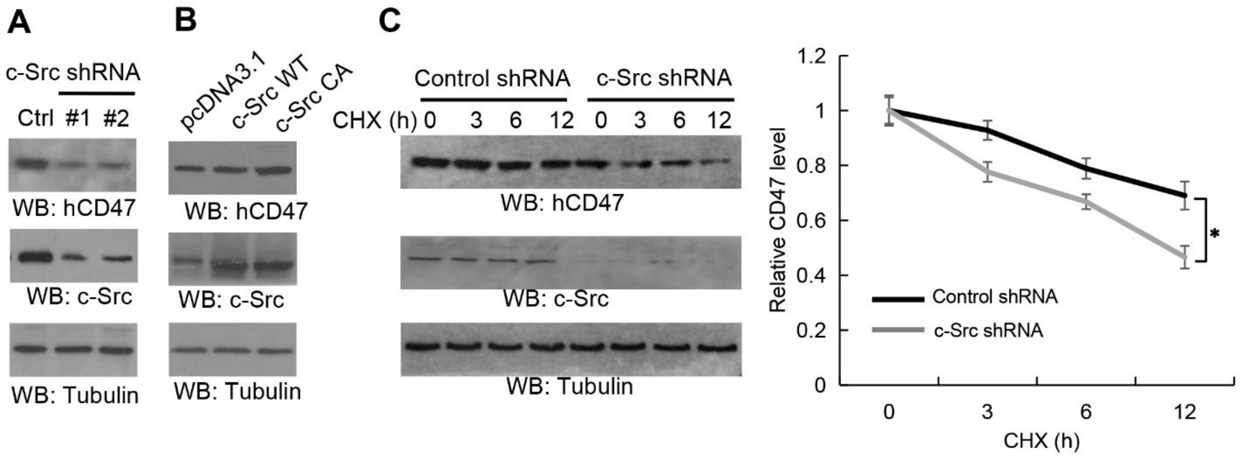

Figure S3. c-Src activation induces CD47 protein upregulation in response to EGF activation.

Immunoblot analyses were performed with the indicated antibodies (A-C).

(A) U87/EGFRvIII cells stably expressed c-Src shRNAs or a control shRNA.

(B) U87/EGFR cells were transfected with control vector, c-Src WT, or c-Src CA for 48 h.

(C) U87/EGFRvIII cells stably expressing c-Src shRNA or control shRNA were treated with CHX (100 μg/ml) for the indicated periods of time. Quantification of relative CD47 protein levels is shown (right panel).

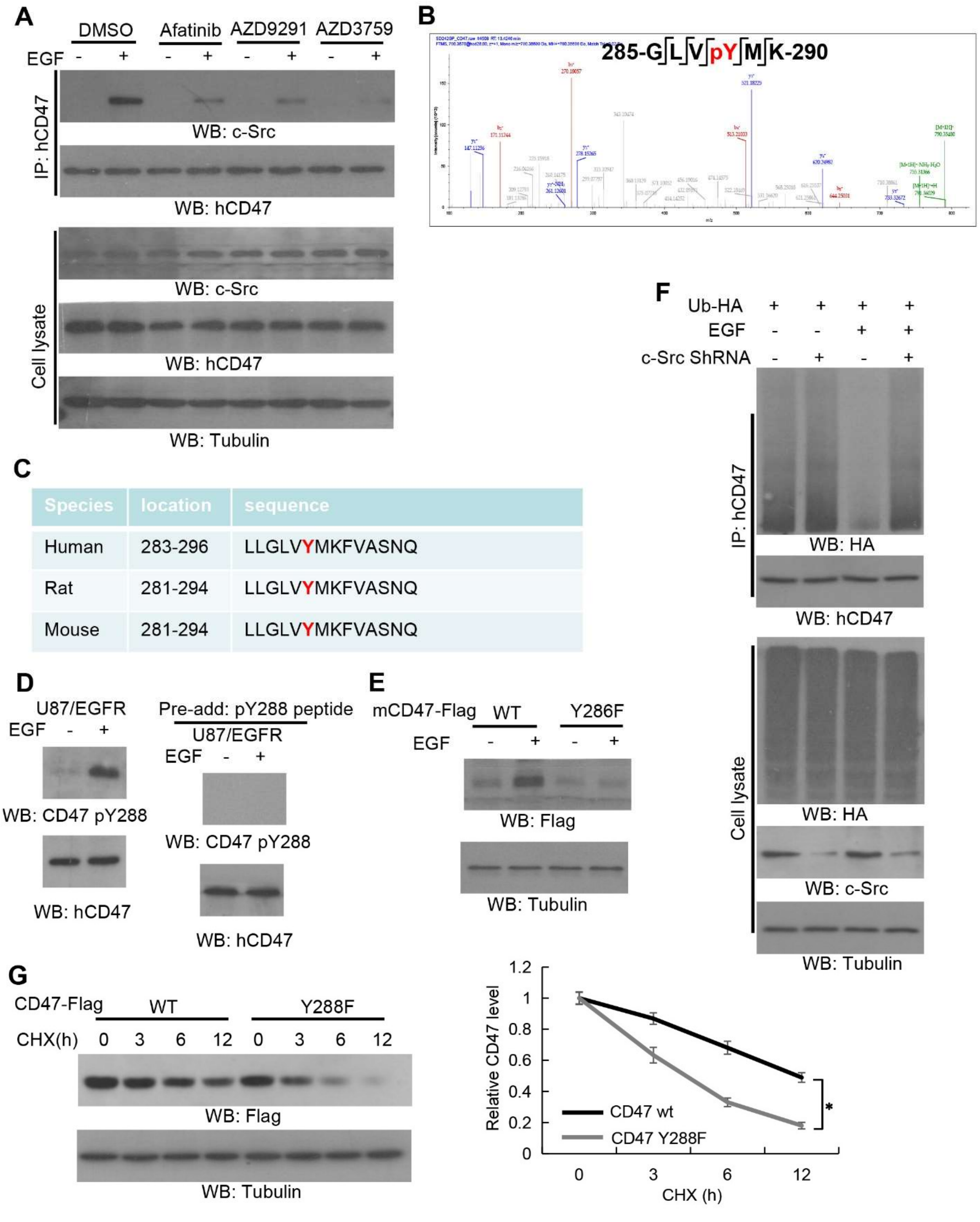

Figure S4. c-Src binds to and phosphorylates CD47 at Y288 and subsequently upregulates CD47 stability by inhibiting CD47 polyubiquitylation.

Immunoblotting analyses were performed with the indicated antibodies (A, D-G).

(A) Serum-starved U251 cells were stimulated with or without EGF (100 ng/ml) for 1 h in the presence or absence of the indicated EGFR inhibitors. Endogenous CD47 was immunoprecipitated.

(B) Serum-starved U251 cells were stimulated with EGF (100 ng/ml) for 1 h, and then endogenous CD47 was immunoprecipitated. Mass spectrometry analysis suggested that Y288 was phosphorylated.

(C) Y288 in the human CD47 protein is conserved in other species. Tyrosine residues are highlighted in red.

(D) Serum-starved U87/EGFR cells were stimulated with or without EGF (100 ng/ml) for 1 h. Immunoblotting analyses were performed with an anti-phospho-CD47 antibody in the presence or absence of specific blocking phosphopeptides.

(E) CD47 knockout CT-2A cells with reconstituted expression of WT Flag-CD47 or Flag-CD47 Y286F were stimulated with or without EGF (100 ng/ml) for 24 h.

(F) U251 cells with stable expression of a c-Src shRNA or a control shRNA were transfected with HA-Ub and then treated with or without EGF (100 ng/ml) for 1 h. MG132 (10 μM) was added to the cells 6 h before they were harvested with guanidine-HCl-containing buffer. Immunoprecipitation was performed with an anti-CD47 antibody.

(G) HEK293T/EGFR cells expressing WT Flag-CD47 or Flag-CD47 Y288F mutant were treated with CHX (100 μg/ml) for the indicated periods of time in the presence of EGF (100 ng/ml). Quantification of relative Flag (CD47) protein levels is shown (left panel).

A

Protein name	Peptide hits
CD47	28
c-Src	17
Keratin	16
Myosin-9	13
MYL12B	9
TRIM21	8

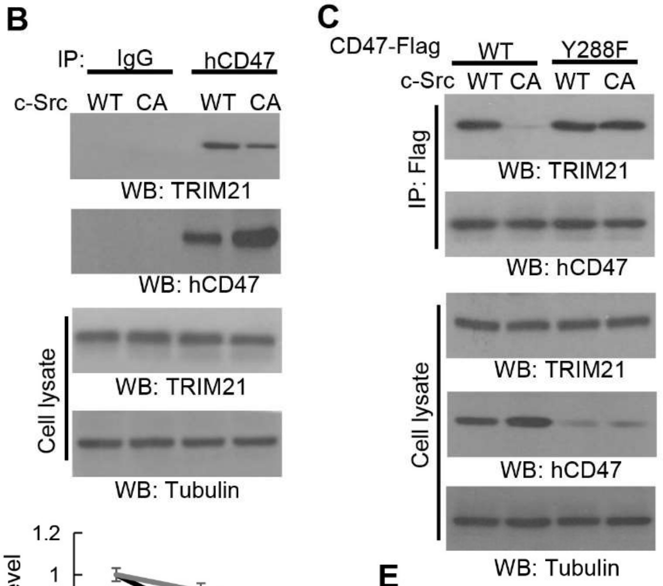

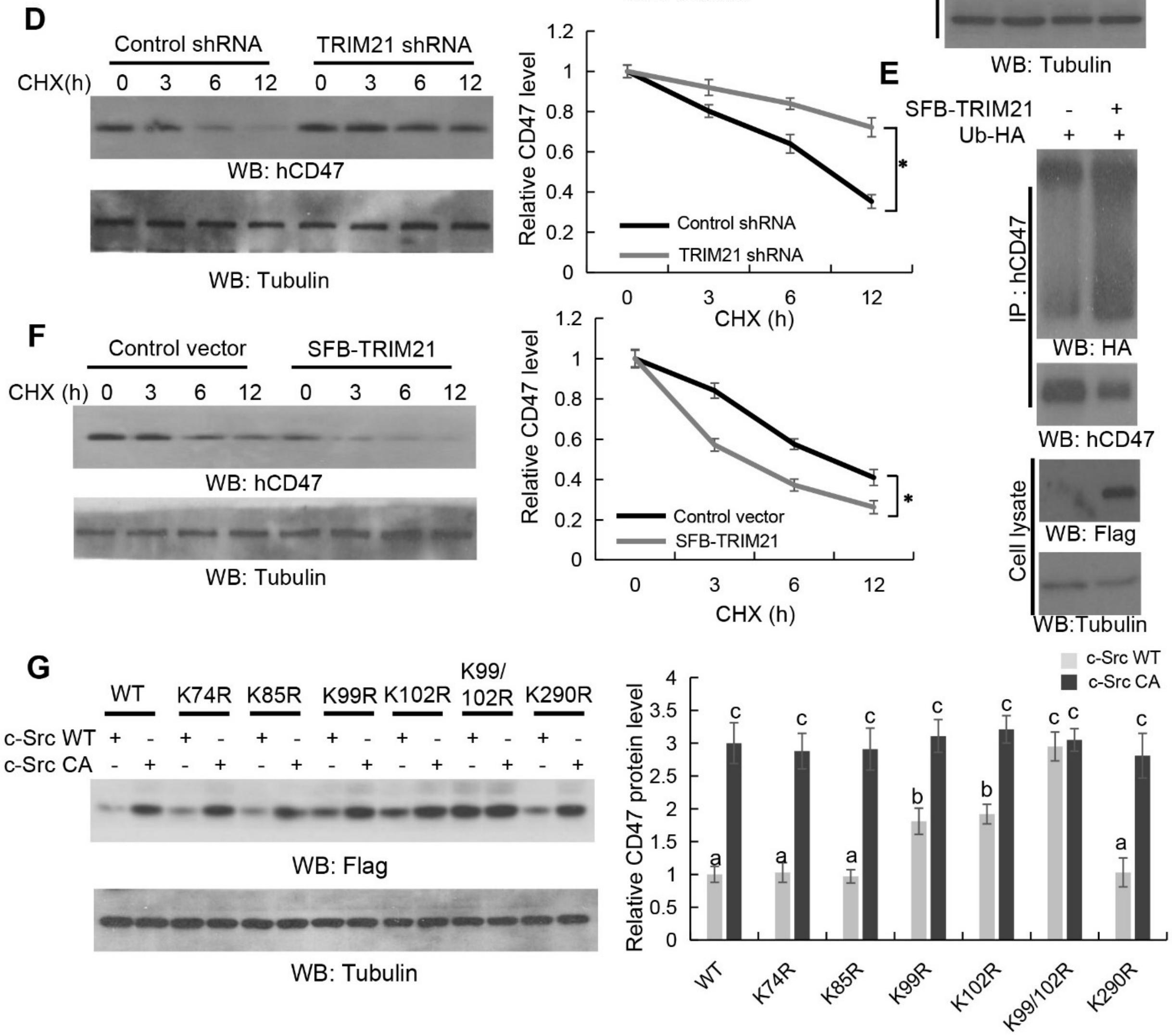

Figure S5. CD47 Y288 phosphorylation inhibits TRIM21-mediated CD47 K99/102

polyubiquitylation and CD47 degradation.

Immunoblotting analyses were performed with the indicated antibodies (B-G).

(A) Selected peptide hits of CD47-associated proteins in U251 cells, as identified by mass spectrometry, are presented.

(B) U251 cells were transfected with c-Src WT or c-Src CA for 48 h. Endogenous CD47 was immunoprecipitated.

(C) HEK293T/EGFR cells were transiently co-transfected with Flag-CD47 or Flag-CD47 Y288F and wild-type c-Src or c-Src CA for 48 h. Immunoprecipitation was performed with anti-Flag beads.

(D) HEK293T cells were transfected with CD47-Flag and TRIM21 shRNA or a control shRNA for 48 h and then treated with CHX (100 μg/ml) for the indicated periods of time. Quantification of relative CD47 protein levels is shown (right panel).

(E) U251 cells were with SFB-tagged TRIM21 and HA-Ub. The cells were harvested with guanidine-HCl-containing buffer. Immunoprecipitation with an anti-CD47 antibody was performed.

(F) HEK293T cells were transfected with Flag-CD47 and SFB-control vector or SFB-TRIM21 for 48 h and then treated with CHX (100 μg/ml) for the indicated periods of time. Quantification of relative CD47 protein levels is shown (right panel).

(G) HEK293T/EGFR cells were co-transfected with WT Flag-CD47 or the indicated Flag-tagged mutants and c-Src WT or c-Src CA for 48 h. Quantification of relative Flag (CD47) protein levels is shown (right panel). The results represent the means ± SD; ANOVA two-way test. Different let

ters indicate significant differences ($p<0.05$).

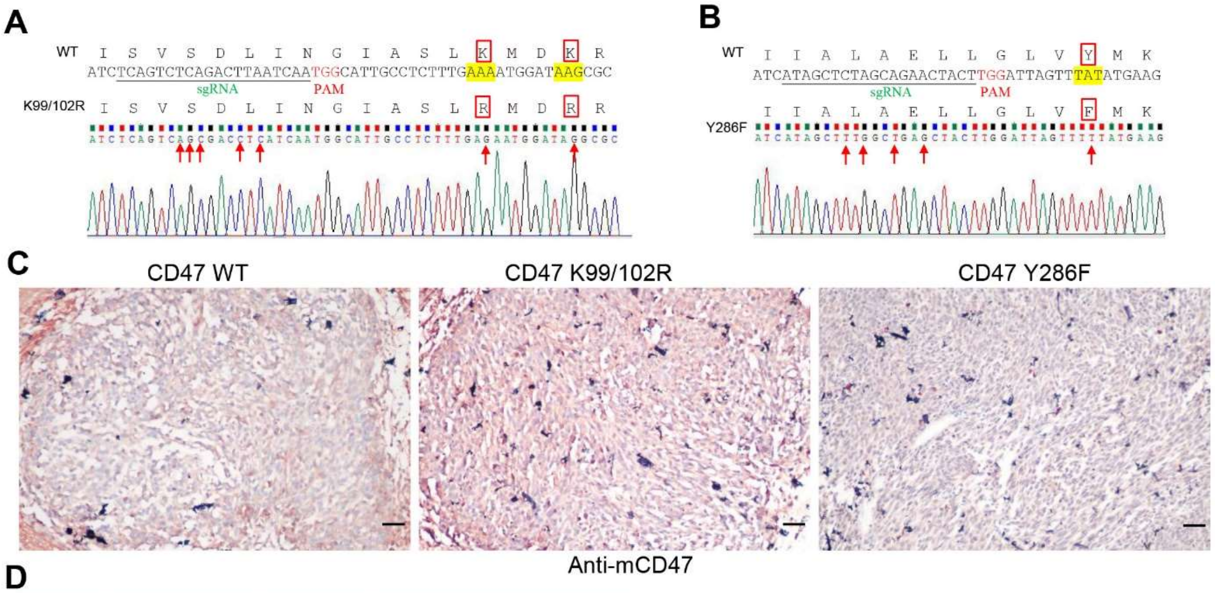

D

	Phagocytic index Field 1	Phagocytic index Field 2	Phagocytic index Field 3	Phagocytic index Field 4
CD47 WT	7	8	5	7
CD47 K99/102R	1	3	1	3
CD47 Y288F	18	18	26	34

	Phagocytic index Field 1	Phagocytic index Field 2	Phagocytic index Field 3	Phagocytic index Field 4
Control	4	9	5	8
α-CD47	14	18	23	26
Afatinib	12	21	26	17
Afatinib+ α-CD47	47	28	40	28

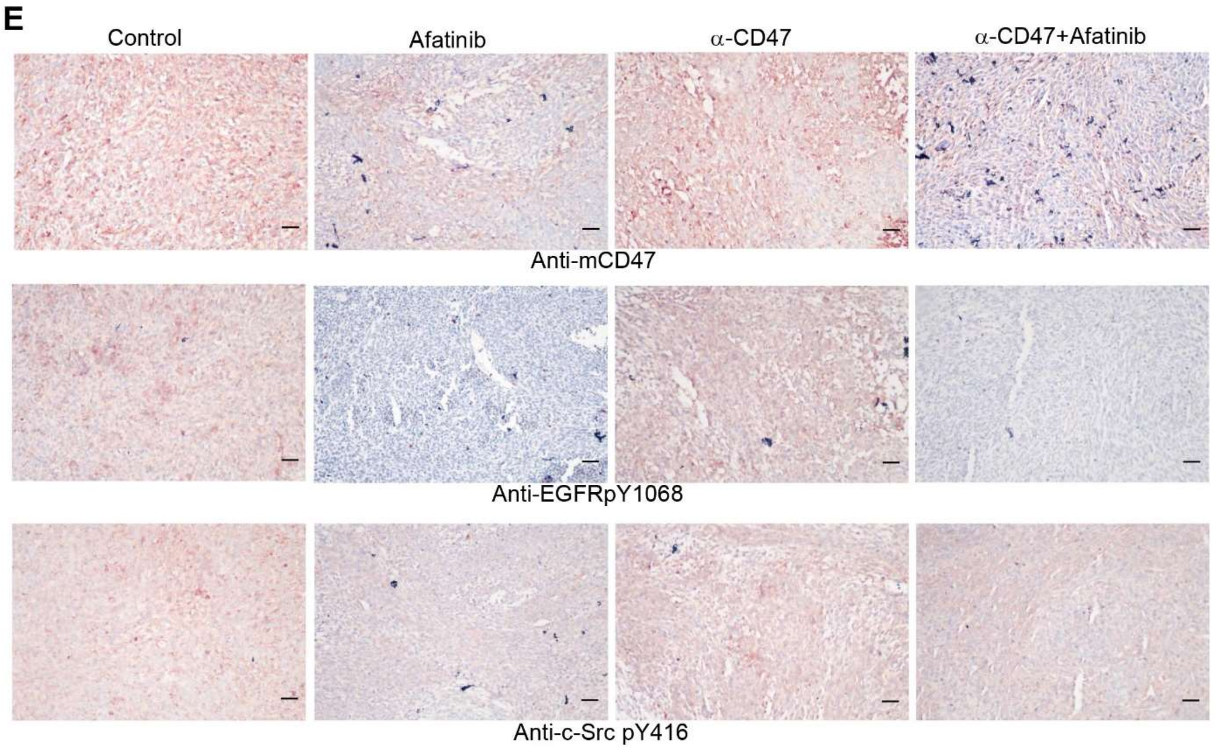

Figure S6. EGFR activation-induced and c-Src-mediated CD47 phosphorylation and

stabilization promote immune evasion of tumor cells and brain tumor growth

(A and B) CRISPR/cas9-mediated knock-in of CD47 Y286F and K99/102R mutants in CT-2A/Luc-GFP cells. The black lines indicate the sgRNA-targeting sequences. The red color highlighted letters indicate the protospacer adjacent motif (PAM). The mutated nucleotides are highlighted by red arrows. The mutated amino acid and its WT counterpart are highlighted by the red box. Silent mutations of the indicated nucleotides were introduced into the sgRNA-targeting sequences to avoid repeat cutting by hSpCas9.

(C) IHC staining of mouse tumor tissues was performed with an anti-CD47 antibody. Representative images are shown. Scale bar, 100 µm.

(D) Immunofluorescent staining of the mouse GBM specimens was performed with the indicated antibodies. The number of macrophages engulfed the cancer cells are shown in the tables.

(E) IHC staining of mouse tumor tissues was performed with the indicated antibodies. Representative images are shown. Scale bar, 100 µm.
